# Novel Prognostic Signatures of Hepatocellular Carcinoma Based on Metabolic Pathway Phenotypes

**DOI:** 10.3389/fonc.2022.863266

**Published:** 2022-05-23

**Authors:** Tingbo Ye, Leilei Lin, Lulu Cao, Weiguo Huang, Shengzhe Wei, Yunfeng Shan, Zhongjing Zhang

**Affiliations:** ^1^ Department of Hepatobiliary Surgery, The First Affiliated Hospital of Wenzhou Medical University, Wenzhou, China; ^2^ Key Laboratory of Diagnosis and Treatment of Severe Hepato-Pancreatic Diseases of Zhejiang Province, The First Affiliated Hospital of Wenzhou Medical University, Wenzhou, China; ^3^ Department of Ultrasound, Wenzhou People’s Hospital, Wenzhou, China; ^4^ Department of Pathology, The Quzhou Affiliated Hospital of Wenzhou Medical University, Quzhou People’s Hospital, Quzhou, China; ^5^ Department of Vascular Surgery, The First Affiliated Hospital of Wenzhou Medical University, Wenzhou, China; ^6^ Department of Hand Surgery and Peripheral Neurosurgery, The First Affiliated Hospital of Wenzhou Medical University, Wenzhou, China

**Keywords:** hepatocellular carcinoma, metabolism, mutant oncogene, tumor immunity, overall survival

## Abstract

Hepatocellular carcinoma is a disastrous cancer with an aberrant metabolism. In this study, we aimed to assess the role of metabolism in the prognosis of hepatocellular carcinoma. Ten metabolism-related pathways were identified to classify the hepatocellular carcinoma into two clusters: Metabolism_H and Metabolism_L. Compared with Metabolism_L, patients in Metabolism_H had lower survival rates with more mutated TP53 genes and more immune infiltration. Moreover, risk scores for predicting overall survival based on eleven differentially expressed metabolic genes were developed by the least absolute shrinkage and selection operator (LASSO)-Cox regression model in The Cancer Genome Atlas (TCGA) dataset, which was validated in the International Cancer Genome Consortium (ICGC) dataset. The immunohistochemistry staining of liver cancer patient specimens also identified that the 11 genes were associated with the prognosis of liver cancer patients. Multivariate Cox regression analyses indicated that the differentially expressed metabolic gene-based risk score was also an independent prognostic factor for overall survival. Furthermore, the risk score (AUC = 0.767) outperformed other clinical variables in predicting overall survival. Therefore, the metabolism-related survival-predictor model may predict overall survival excellently for HCC patients.

## Introduction

Hepatocellular carcinoma (HCC) is one of the most prevalent primary cancers worldwide and ranks third in all cancer-related mortality (1, 2). Numerous therapeutic strategies for treating liver cancer have been developed, including surgical resection, radiofrequency ablation, liver transplantation, and targeted therapy (3, 4). Despite the fact that great progress has been made in clinical treatment, the survival rates of liver cancer patients within 5 years are still as low as 18% because of the highly malignant tumors, the high recurrence rate, and drug resistance (5, 6). Several factors have been identified to affect and predict HCC prognosis, such as microRNAs and blood groups (7, 8). However, these factors still could not predict the prognosis accurately. Therefore, it is paramount to exploring how specific cellular tumor progression pathways contribute to HCC prognostic stratification for cancer treatment development.

It is proposed that cancer cells must modify their metabolic programs to obtain energy and macronutrient during rapid growth (9, 10). Metabolism regulated by oncogenes allow tumor cells to survive and proliferate in the tumor microenvironment (11). In fact, metabolic reprogramming is a well-established hallmark of cancer (12, 13). Many studies suggested that in order to adapt to the growth and proliferation of HCC cells, the aberrant metabolism of cells develops, which is related to the prognosis of patients (14–16). Strikingly, the functional importance of metabolic alterations often diverges on tumor subtypes, leading to visible therapeutic vulnerability discrepancies in cancer therapy (17, 18). However, metabolic heterogeneity within different HCC subtypes defined by distinct metabolic pathways, which may further result in differences in oncogenes and tumor immunity, has not been well implemented.

**Figure 1:**
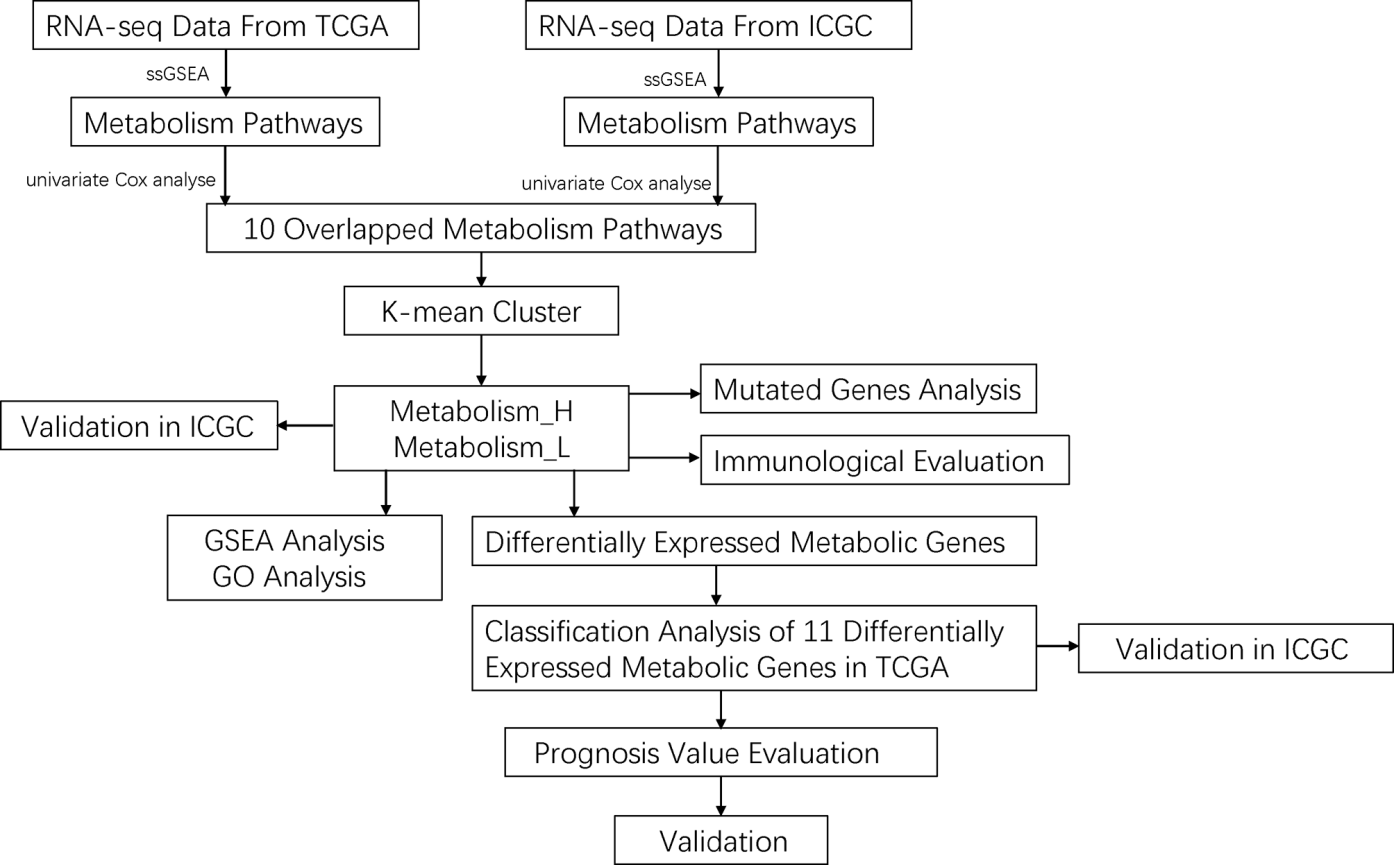
The workflow of this work.

In this study, we classified HCC into two different clusters by metabolism-related pathways profiling: Metabolism_H and Metabolism_L. Then, we explored the relationship between the classification and mutation of oncogenes and tumor immunity. Differentially expressed metabolic genes (DEMGs) were identified according to metabolism status and the DEMG-based survival-predictor model was also developed for predicting survival rates of HCC patients, as shown in **Figure 1**. Moreover, immunohistochemistry staining showed that, compared with normal tissues, 8 of the 11 genes were differentially expressed in cancer tissues, while 3 genes revealed no significant differences. Most of these differentially expressed genes (DEGs) were consistent with our prognostic model, which further verified the reliability of the model. Therefore, the DEMG-based survival-predictor model might have the great potential to predict survival rates of HCC patients.

## Materials and Methods

### Patient Datasets

The data of RNA-seq and clinical features in HCC patients were extracted from The Cancer Genome Atlas (TCGA) (https://portal.gdc.cancer.gov/) and LIRI_JP from International Cancer Genome Consortium (ICGC) (https://icgc.org/). For further analysis, a total of three hundred and twelve patients with both gene expression and overall survival (OS) data were extracted. Moreover, a total of thirty pairs of paraffin-embedded specimens were collected for this study from the pathology department, including both HCC and matched para-carcinoma tissues. All the specimens were obtained from HCC patients who underwent tumor resection. Clinical and pathological information of these specimens are presented in **Table S1**. This study was approved by the Ethics Department of the First Affiliated Hospital of Wenzhou Medical University.

### Clustering

We first downloaded KEGG pathways from GSEA (http://software.broadinstitute.org/gsea/index.jsp). Then, we conducted univariate Cox analyses by SPSS 19.0 in TCGA and LIRI_JP, respectively, to acquire significant metabolism-related pathways. We selected the 10 mutual metabolism-related pathways between TCGA and LIRI_JP. For each HCC dataset, we quantified the enrichment levels of the 10 mutual metabolism-related pathways in each HCC sample through the single-sample gene set enrichment analysis (ssGSEA) score. Based on the enrichment levels (ssGSEA scores) of the 10 mutual metabolism-related pathways, we performed hierarchical clustering analysis of HCC.

### Multi-Omics Analysis

We obtained the mutation data of HCC patients from TCGA and LIRI_JP. The data containing somatic variants were stored in the form of Mutation Annotation Format (MAF). Mutation data were analyzed and summarized using “GenVisR” package in R software. Copy number variation (CNV) analysis was performed using data of TCGA through cBioPortal (www.cbioportal.org).

To screen out methylation-driven genes, we calculated correlation between gene methylation level and expression using the “MethylMix” package in R software with corFilter = −0.3 and adjusted *p*-value < 0.05 as the cutoff value. Then, based on the median of beta-values of methylation, we divided HCC patients into two groups and performed Kaplan–Meier (K-M) survival analysis. The genes were considered to be significantly associated with OS based on the threshold of *p* < 0.05.

### Evaluation of Immune Cell Infiltration Level, Tumor Purity, and Stromal Content Between the Two Clusters

We used ESTIMATE (19) to evaluate the immune cell infiltration level (immune score), tumor purity, and stromal content (stromal score) of each HCC sample in Metabolism_H and Metabolism_L cohorts.

### Assessment of Tumor-Infiltrating Immune Cells

After removing data with *p*-value >0.05 of the correlation between the samples and the immune cells, the RNA-sequencing data of TCGA and LIRI_JP were used to estimate the proportions of 22 types of infiltrating immune cells using the CIBERSORT algorithm following the procedure as previously reported (20). We calculated the expression level of 22 immune cells in each sample through a deconvolution algorithm to quantify the number of cells in each sample. The R packages of e1071, parallel, and preprocessCore were used. The estimate package could adopt the RNA-seq data to calculate the immune and mechanism scores of the specimen, and then evaluate the purity of the tumor. The principle was to evaluate the above content through the signature of the characteristic tumor’s RNA-seq and the input file needed RNA-seq. Common genes data were also required to calculate the inner matrix. We used the estimate package to calculate the score of each sample’s immunity and matrix, then obtained the purity of the tumor and used it for the next calculation.

### Identification of Metabolism-Related Pathways–Immune-Related Pathways Networks

We first quantified the enrichment levels of immune-related pathways in each HCC sample of Metabolism_H and Metabolism_L by the ssGSEA score. Then, metabolism-related pathways–immune-related pathways networks were drawn by cytoscape online software (http://www.cytoscape.org/).

### Gene Set Enrichment and Functional Enrichment Analyses

We performed gene set enrichment analysis of the LIRI_JP and TCGA datasets by GSEA (R implementation) (21, 22). This analysis identified the KEGG (22) pathways that were upregulated or downregulated in Metabolism_H and Metabolism_L, respectively. Terms in KEGG with a false discovery rate (FDR) < 0.05 were considered significantly enriched and were visualized using R package “plyr”, “grid”, “gridExtra”, and “ggplot2” (23). Gene ontology (GO) analysis was performed using the R package “GOplot” (FDR < 0.05) (24).

### DEMG-Based Classifiers for Overall Survival

The least absolute shrinkage and selection operator (LASSO)-Cox regression model (25) was used to identify the most accurate predictive DEMGs for OS. The risk score of each patient was determined based on the DEMG-based classifiers. The patients were categorized into two groups by median score. The survival estimation of patients was analyzed by the K-M method.

### Predictive Performance of the DEMG-Based Classifiers

The univariate and multivariate Cox regression analyses were conducted to identify significant prognostic predictors associated with OS. The time-dependent receiver operating characteristics (tdROC) analysis by the “timeROC” package of R software was used to assess performance of clinical variables and classifiers. The area under the curve (AUC) of tdROC represented the predictive accuracy. In addition, *p*-values < 0.05 were considered statistically significant.

### Immunohistochemical Staining

Paraffin-embedded liver tissue sections (4 µm) were deparaffinized in xylene and rehydrated in ethanol solutions. Then, the tissue sections were boiled in sodium citrate buffer using a microwave oven for 15 min to perform antigen retrieval, and 3% hydrogen peroxide was used for inhibiting the activity of endogenous peroxidase. Subsequently, to prevent nonspecific binding, the sections were blocked with 5% normal goat serum for 30 min at a temperature of 37°C. Primary antibodies for RRM2 (Proteintech, Wuhan, China, 11661-1-AP; 1:200), LPCAT1 (Proteintech, Wuhan, China, 16112-1-AP; 1:200), G6PD (Proteintech, Wuhan, China, 25413-1-AP; 1:500), CYP3A5 (Proteintech, Wuhan, China, 13737-1-AP; 1:200), CYP2C9 (Proteintech, Wuhan, China, 16546-1-AP; 1:500), BDH1 (Proteintech, Wuhan, China, 15417-1-AP; 1:200), ADH4 (Proteintech, Wuhan, China, 16474-1-AP; 1:200), PYCR1 (ABclonal, Wuhan, China, A13346; 1:100), PTGES (ABclonal, Wuhan, China, A18632; 1:100), HK2 (ABclonal, Wuhan, China, A0994; 1:100), and ADH1C (ABclonal, Wuhan, China, A8081; 1:100) diluted with antibody diluent buffer were added to corresponding tissues and then incubated overnight at 4°C. After washing, secondary antibodies (goat anti-rabbit antibody) (BioSharp Inc., China) were employed to incubate for 1 h. Finally, the sliders were stained with diaminobenzidine (DAB) (Beijing Zhongshan Golden Bridge Biotechnology, China) for color visualization and counterstained with hematoxylin. The staining results were evaluated by three blinded pathologists independently, and their median values were adopted as final score. Staining intensity was divided into four levels according to the following criteria: 0 point (negative), 1 point (weak), 2 points (moderate), and 3 points (strong). The proportion of positive staining area was scored as follows: 0 point (<5%), 1 point (5%–25%), 2 points (26%–50%), 3 points (51%–75%), and 4 points (>75%). The total scores consisted of multiplying the positive staining area percentage scores by staining intensity scores (26, 27).

### Data Analysis

The Student’s *t*-test, Wilcoxon test, and other data processing were completed by SPSS 19.0 and GraphPad Prism 7.0 software. The K-M log rank test was calculated by medcalc (Version 19.0). Continuous variables were expressed as the mean ± standard deviation (SD). When all the hypotheses have a *p*-value < 0.05, the difference is statistically significant.

## Results

### Metabolism-Related Pathways Profiling Identified Two HCC Clusters

**Figure 2:**
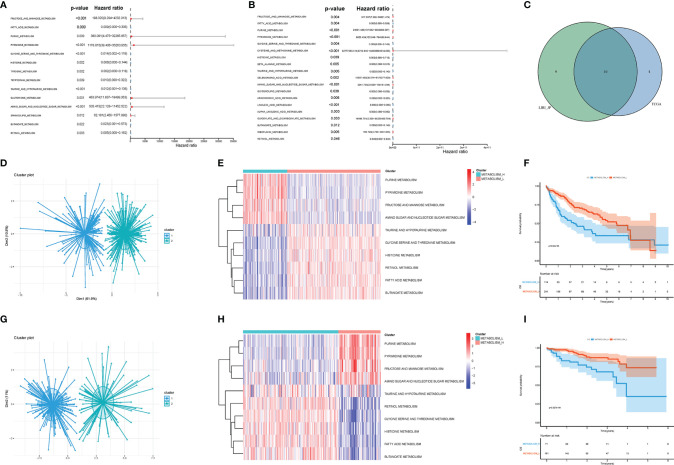
Metabolism-related pathways profiling identified two HCC clusters. **(A)** In TCGA, univariate Cox analyses showed that there were 14 significant metabolic pathways contributing to OS in HCC. **(B)** In LIRI_JP, there were 19 significant pathways. **(C)** A Venn diagram showed 10 metabolism-related pathways shared by TCGA and LIRI_JP. **(D, E)** Based on the different gene expressions of the 10 pathways, HCC was clustered: Metabolism_H and Metabolism_L in TCGA. **(G, H)** HCC was also clustered: Metabolism_H and Metabolism_L in LIRI_JP; Kaplan–Meier survival analysis results indicated that the two clusters had significantly different survival rates in both **(F)** TCGA (log-rank test *p*-value = 6.02e−05) and **(I)** LIRI_JP (log-rank test *p*-value = 2.527e−04). HCC, hepatocellular carcinoma; OS, overall survival.

Firstly, we acquired KEGG signaling pathways by GSEA and singled out 42 metabolism-related pathways (**Table S2**). There were 14 significant pathways in TCGA (**Figure 2A**) and 19 significant pathways in LIRI_JP (**Figure 2B**), which were both associated with OS of HCC. Among the metabolism-related pathways, there were 10 pathways shared by the two datasets (**Figure 2C**).

Next, the 10 metabolism-related pathways were clustered in TCGA and LIRI_JP, respectively (**Figures 2D, G**). Interestingly, the two datasets showed similar clustering results, with two clusters being clearly separated (**Figures 2E, H**). The 10 metabolic pathways related to prognosis were clustered using the k-means method. As shown in **Figure 2E**, in the two classifications, 10 pathways have different expression trends. We defined the one with poor prognosis as Metabolism_H, and the other as Metabolism_L. The clusters significantly correlated with histologic grade, TMN stage, and AJCC pathological stage (*p* < 0.001) (**Table 1**). Moreover, survival analyses showed that the different metabolic subtypes of HCC had distinct clinical outcomes. The Metabolism_L subtype likely had a better survival prognosis than the Metabolism_H subtype (**Figures 2F, I**).

**Table 1:** Correlations between risk score of the metabolism-related pathways classifier with overall survival and clinicopathological characteristics in the TCGA-LIHC cohort.

Clinicopathological variables	Number of patients (*n* = 355)	Metabolism_H	Metabolism_L	*p*-value
Age				
<65 (*n*, %)	210 (59.2%)	78 (37.1%)	132 (62.9%)	
≥65 (*n*, %)	145 (40.8%)	36 (24.8%)	109 (75.2%)	0.015
Gender				
Male (*n*, %)	239 (67.3%)	71 (29.7%)	168 (53.7%)	
Female (*n*, %)	116 (32.7%)	43 (37.1%)	73 (62.9%)	0.164
Histologic Grade				
G1+G2 (*n*, %)	219 (61.7%)	50 (22.8%)	169 (77.2%)	
G3+G4 (*n*, %)	131 (36.9%)	63 (48.1%)	68 (51.9%)	<0.001
NA	5(1.4%)			
TNM staging system				
T1+T2 (*n*, %)	262 (73.8%)	70 (26.7%)	192 (73.3%)	
T3+T4 (*n*, %)	91 (25.6%)	44 (48.4%)	47 (51.6%)	<0.001
NA	2(0.6%)			
N0 (*n*, %)	244 (68.7%)	84 (34.4%)	160 (65.6%)	
N1 (*n*, %)	2 (0.6%)	1 (50.0%)	1(50.0%)	<0.001
NA	109(30.7%)			
M0 (*n*, %)	256 (72.1%)	92 (35.9%)	164 (64.1%)	
M1 (*n*, %)	4 (1.1%)	2 (50.0%)	2 (50.0%)	<0.001
NA	95(26.8%)			
AJCC pathological stage				
I+II (*n*, %)	246 (69.3%)	65 (26.4%)	181 (73.6%)	
III+IV (*n*, %)	88 (24.8%)	43 (48.9%)	45 (51.1%)	<0.001
NA	21 (5.9%)			

NA, not available.

### The Mutant Oncogenes, Copy Number Variation, and DNA Methylation Analysis

**Figure 3:**
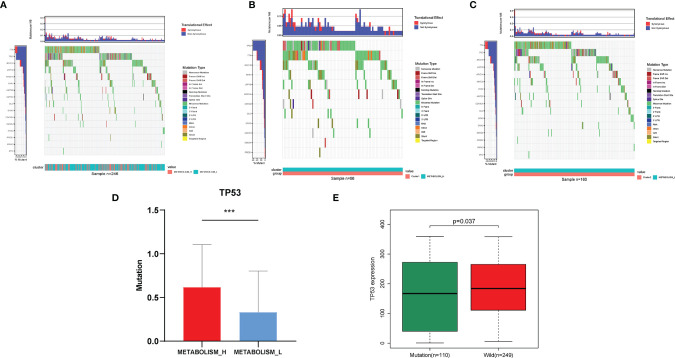
Different mutant oncogenes between HCC Metabolism_H and Metabolism_L. **(A)** Waterfall plots suggested that *TP53*, *TTN*, and *MUC16* were main mutant oncogenes in HCC patients, but they were different between **(B)** Metabolism_H and **(C)** Metabolism_L. **(D)** Metabolism_H was more likely to have *TP53* mutations (Student’s *t*-test, *p* < 0.001). **(E)** The mutated *TP53* gene was less expressed than the wild type (Wilcoxon test, *p* = 0.037). HCC, hepatocellular carcinoma. *p < 0.05, **p < 0.01, ***p < 0.001.

To explore why the Metabolism_L subtype likely had a better survival prognosis than the Metabolism_H subtype, we analyzed three parts of the differences between the two clusters: the mutant oncogenes, copy number variation, and DNA methylation. Obviously, there were several oncogenes mutated in most HCC patients, especially *TP53*, *TTN*, and *MUC16* mutated in more than 50 samples (20%) (**Figures 3A–C**). For most cancer types, the *TP53, TTN*, and *MUC16* genes were found to mutate frequently (25). The waterfall plot illustrated that *TP53* and *TTN* were mutated in different numbers of patients. Metabolism_H was more likely to have *TP53* mutations (Student’s *t*-test, *p* < 0.001) (**Figure 3D**), while *TTN* showed the opposite. **Figure 3E** shows that the mutated *TP53* gene was less expressed than the wild (Wilcoxon test, *p* < 0.05).

As demonstrated in **Figure S1**, HCC patients showed significantly different CNV in the two subtypes. For instance, according to the blue representing deletions and the red representing amplifications, large chunks of DNA were removed in chromosome 4 of the Metabolism_H subtype. The detailed information of CNV of each gene is shown in **Table S3**. **Figure S1C** shows ten genes with the most obvious differences in CNV between Metabolism_H and Metabolism_L: NUP210L, KCNN3, FAM189B, SCAMP3, CTSS, DPM3, EFNA1, GBA, GBAP1, and KRTCAP2, all of which were amplified on chromosome 1, and more frequent in Metabolism_H.

**Figure 4:**
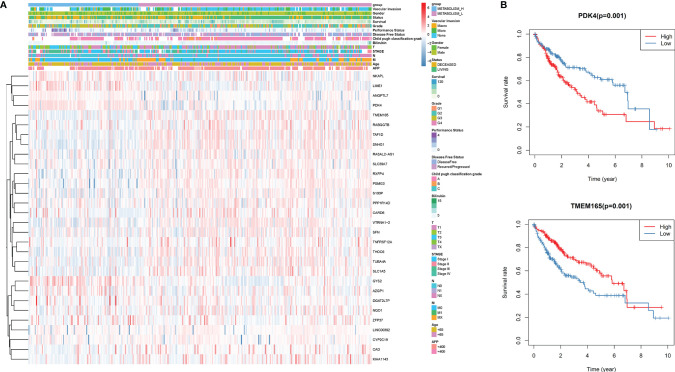
The DNA methylation level analysis. **(A)** The heatmap of 30 methylation driving genes associated with prognosis. **(B)** K–M survival analysis of HCC patients with hypermethylated or hypomethylated PDK4 and TMEM165.

Through whole-genome DNA methylation analysis, we firstly screened out 240 methylation-driven genes whose genetic expressions were negatively correlated with methylation, and found that 30 of them were related to the HCC prognosis. Then, based on the 30 methylation-driven genes, the heatmap indicated that methylation levels of the 30 genes were significantly different between Metabolism_H and Metabolism_L (**Figure 4A**). For example, the methylation level of PDK4 was higher in Metabolism_H, while the methylation level of TMEM165 was higher in Metabolism_L. Moreover, the survival analyses also showed that the survival rates of HCC patients with hypermethylated PDK4 and hypomethylated TMEM165 were lower, which was consistent with differences in gene methylation levels and prognosis between Metabolism_H and Metabolism_L (**Figure 4B**).

### Immunological Evaluation of the Metabolism-Related Pathway-Based HCC Subtypes

Due to the fact that tumor infiltration lymphocytes were closely related to metabolism and prognosis of tumors (28), we explored the immune infiltration of the metabolism-related pathway-based HCC subtypes according to immune scores (**Figure S2**). When comparing the tumor immunity microenvironment of the two HCC subtypes, we found that the two clusters were significantly different. Compared with Metabolism_L, the immune scores were significantly higher in Metabolism_H (Kruskal–Wallis test, *p* < 0.001) (**Figure S2B**). In addition, although stromal scores did not have significant difference between Metabolism_H and Metabolism_L in TCGA, we obtained opposite trends when comparing the tumor purities and stromal scores of the two HCC subtypes. The tumor purity was higher in Metabolism_L while the stromal score was higher in Metabolism_H (Kruskal–Wallis test, *p* < 0.05) (**Figures S2A, C**). The ESTIMATE Score was also higher in Metabolism_H (Kruskal–Wallis test, *p* < 0.01) (**Figure S2D**). In conclusion, these results indicated that Metabolism_H contained more immune cells and stromal cells, while Metabolism_L contained more tumor cells.

**Figure 5:**
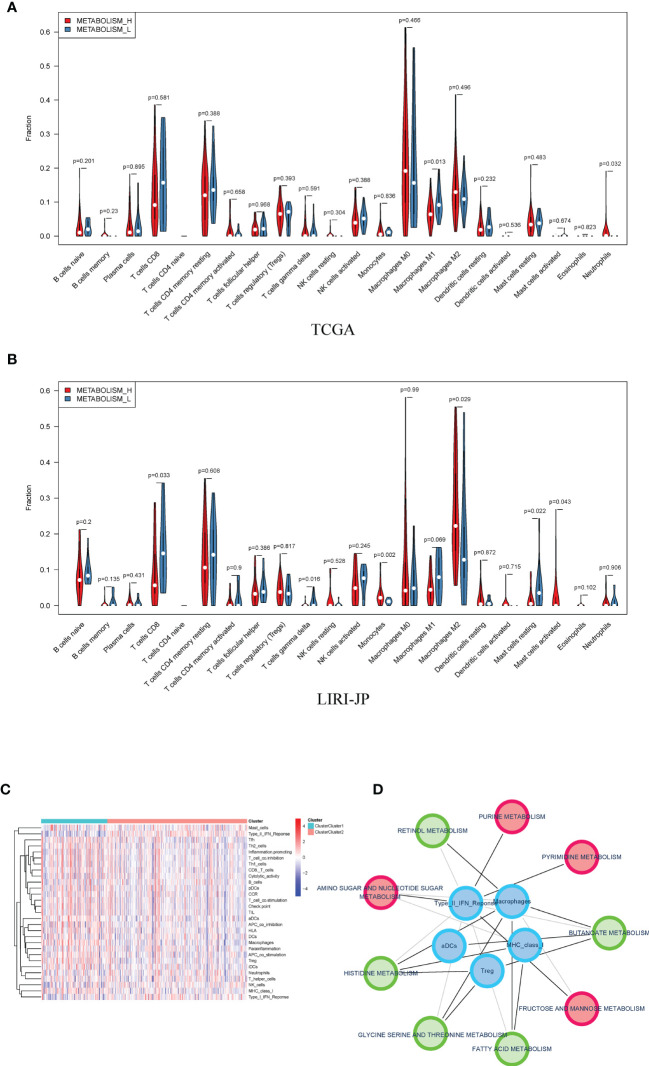
Distribution of immune cells in Metabolism_H and Metabolism_L. **(A)** In TCGA, Metabolism_L contained more naive B cells, gamma delta T cells, and resting mast cells (Wilcoxon test), the same as in **(B)** LIRI−JP. **(C)** A heatmap showed that different clusters led to significantly different gene expressions in immune pathways. **(D)** A metabolism-related pathways–immune-related pathways network indicated that metabolic pathways were associated with tumor immunity.

Therefore, we analyzed immune cell makeups of the two subtypes and found that they were obviously different. Metabolism_H contained more M2 macrophages in LIRI_JP and neutrophils in TCGA, while Metabolism_L contained more CD8 T cells in LIRI_JP and M1 macrophages in TCGA (Wilcoxon test, *p* < 0.05) (**Figures 5A, B**). Based on the two subtypes, we analyzed their immune pathways, which were also differently expressed (**Figure 5C**). Then, for identifying the relationship between metabolism and immune in HCC, a metabolism-related pathways–immune-related pathways network was conducted including 5 immune-related pathways and 9 metabolism-related signal pathways (**Figure 5D**).

Furthermore, we further analyzed the immune checkpoint-related gene expression and found that the expression levels of PD-L1, CTLA-4, TIM-3 were significantly higher in Metabolism_H (**Figure S3A**). Based on the previous study of the cancer stemness (29), mRNA expression-based stemness index (mRNAsi) was higher in Metabolism_H (**Figure S3B**).

### GSEA-Based KEGG Analysis and GO Analysis

**Figure 6:**
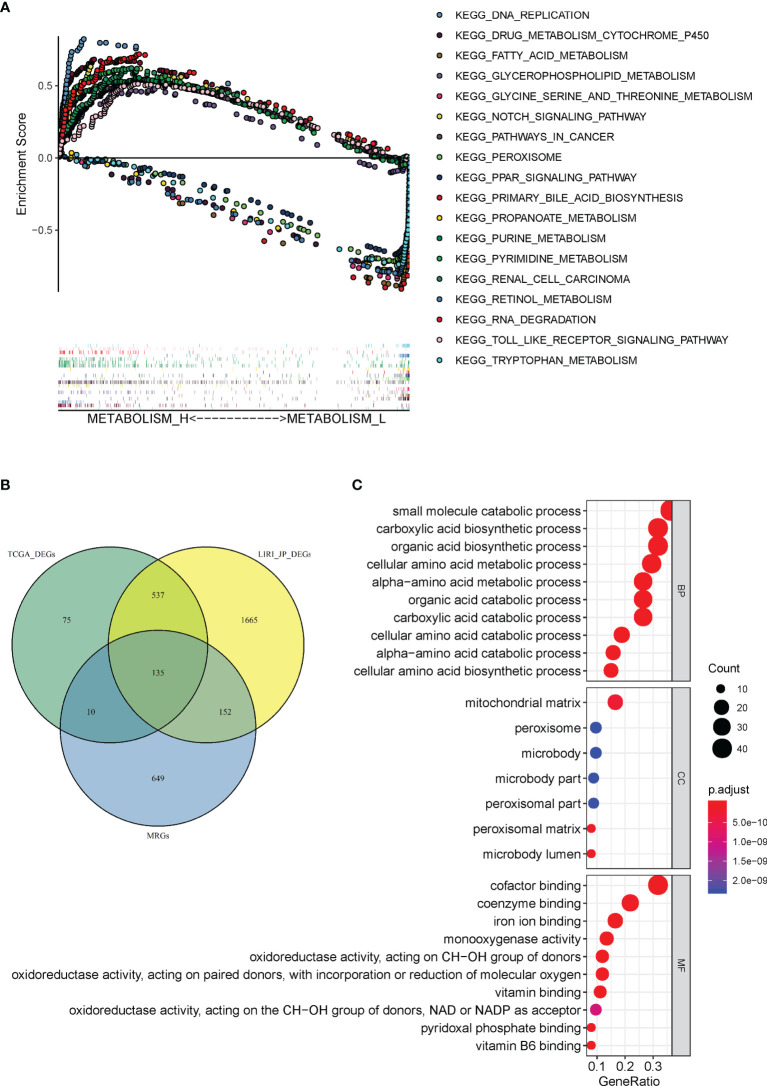
Gene set enrichment and functional enrichment analyses. **(A)** KEGG pathways enriched in Metabolism_H and Metabolism_L, respectively. **(B)** A Venn diagram showed 135 DEMGs were overlapped among TCGA_ DEGs, LIRI_JP_DEGs, and MRGs. **(C)** KEGG pathways in Immunity_H and Immunity_L.

A total of 110 prominent KEGG pathways including pathways expressed differently in Metabolism_H and Metabolism_L were selected (**Table S4**). The 82 high-expression pathways in Metabolism_H, such as “Pathways in cancer” and “TOLL like receptor signaling pathways”, were related to tumor proliferation and metastasis, indicating worse survival prognosis of Metabolism_H. However, the 28 high-expression pathways in Metabolism_L were mainly concentrated on metabolic process, such as “Tryptophan metabolism”, “Primary bile acid biosynthesis”, and “Retinol metabolism”. **Figure 6A** shows GSEA enrichment plots of representative gene sets on several representative pathways of Metabolism_H and Metabolism_L.

There were 946 metabolism-related genes (MRGs) that expressed in 110 prominent KEGG pathways. There were 757 DEGs between Metabolism_H and Metabolism_L in TCGA and 2,468 DEGs in LIRI_JP. A total of 135 DEMGs overlapped among TCGA_DEGs, LIRI_JP_DEGs, and MRGs in the Venn diagram (**Figure 6B**). The 135 DEMGs were mainly associated with the following biological processes: small molecule catabolic process; carboxylic acid biosynthetic and catabolic processes; organic acid biosynthetic and catabolic process; cellular amino acid biosynthetic, metabolic, and catabolic process; and alpha-amino acid metabolic and catabolic process (**Figure 6C**). The results also indicated that the DEMGs were mainly associated with the following cellular contents: mitochondrial matrix, peroxisome, and microbody (**Figure 6C**). Moreover, the DEMGs were related to molecular functions, such as coenzyme binding, iron ion binding, and cofactor binding (**Figure 6C**).

### Prognostic Value of DEMGs

**Figure 7:**
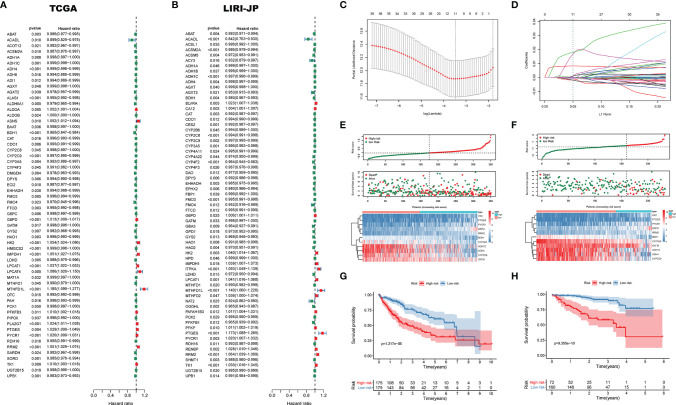
The survival-predictor model based on eleven DEMGs. Univariate Cox analyses showed that 60 DEMGs contributed to the OS in **(A)** TCGA and **(B)** LIRI_JP, respectively. **(C, D)** The LASSO regression model identified the 11 most accurate predictive DEMGs in TCGA. **(E)** HCC patients were divided into two groups by the median risk score cutoff point in TCGA: high risk and low risk. **(F)** According to the same cutoff point, HCC patients were also divided into two groups in LIRI_JP; Kaplan–Meier Survival analysis results indicated that the two groups had significantly different survival rates in both **(G)** TCGA (log-rank test *p*-value = 1.217e−06) and **(H)** LIRI_JP (log-rank test *p*-value = 9.355e−10). DEMGs, differentially expressed metabolic genes; HCC, hepatocellular carcinoma.

In order to investigate the effect of DEMGs on HCC prognosis, we first conducted univariate Cox analyses in TCGA and LIRI_JP, respectively. Sixty DEMGs in TCGA and LIRI_JP related to mortality were identified (**Figures 7A, B**). Among the 60 DEMGs, there are 36 genes shared by the two datasets (**Figure 7C**). According to the results of the LASSO-Cox regression model, 11 prognostic DEMGs with non-zero regression coefficients were finally chosen as the potential prognostic biomarkers for the OS of HCC patients (**Figures 7C, D**). The detailed information of DEMGs for constructing the prognostic signature is summarized in **Table S5**. The formula of the eleven-DEMG survival-predictor model was as follows: eleven-DEMG survival-predictor model score = (0.0177074543570851 * RRM2) + (0.000599168151290748 * PYCR1) + (0.000451238392995456 * PTGES) + (0.0000832126857397287 * LPCAT1) + (0.0154490143978134 * HK2) + (0.00824505291131197 * G6PD) − (0.00170896754573046* CYP3A5) − (0.000591128394736733* CYP2C9) − (0.00190395464082525 * BDH1) − (0.000171745083443705 * ADH4) − (0.000267396449305932 * ADH1C). Based on the survival-predictor model, we evenly divided HCC patients into two groups by the median risk score cutoff point, whose value is −0.0687, in TCGA: high risk and low risk (**Figure 7E**). The enrichment levels of the 11 genes in the two groups quantified by the ssGSEA was also significantly different. Then, K-M analysis showed that survival rates were significantly lower in the high-risk group (*p* < 0.001) (**Figure 7G**). Interestingly, we used the same eleven-DEMG survival-predictor model and cutoff point to cluster patients in LIRI_JP, in which the similar results were obtained (**Figure 7F**). The survival analysis also indicated that high risk had a worse OS (*p* < 0.001) (**Figure 7H**).

### Differential Expression Levels of the Eleven DEMGs

**Figure 8:**
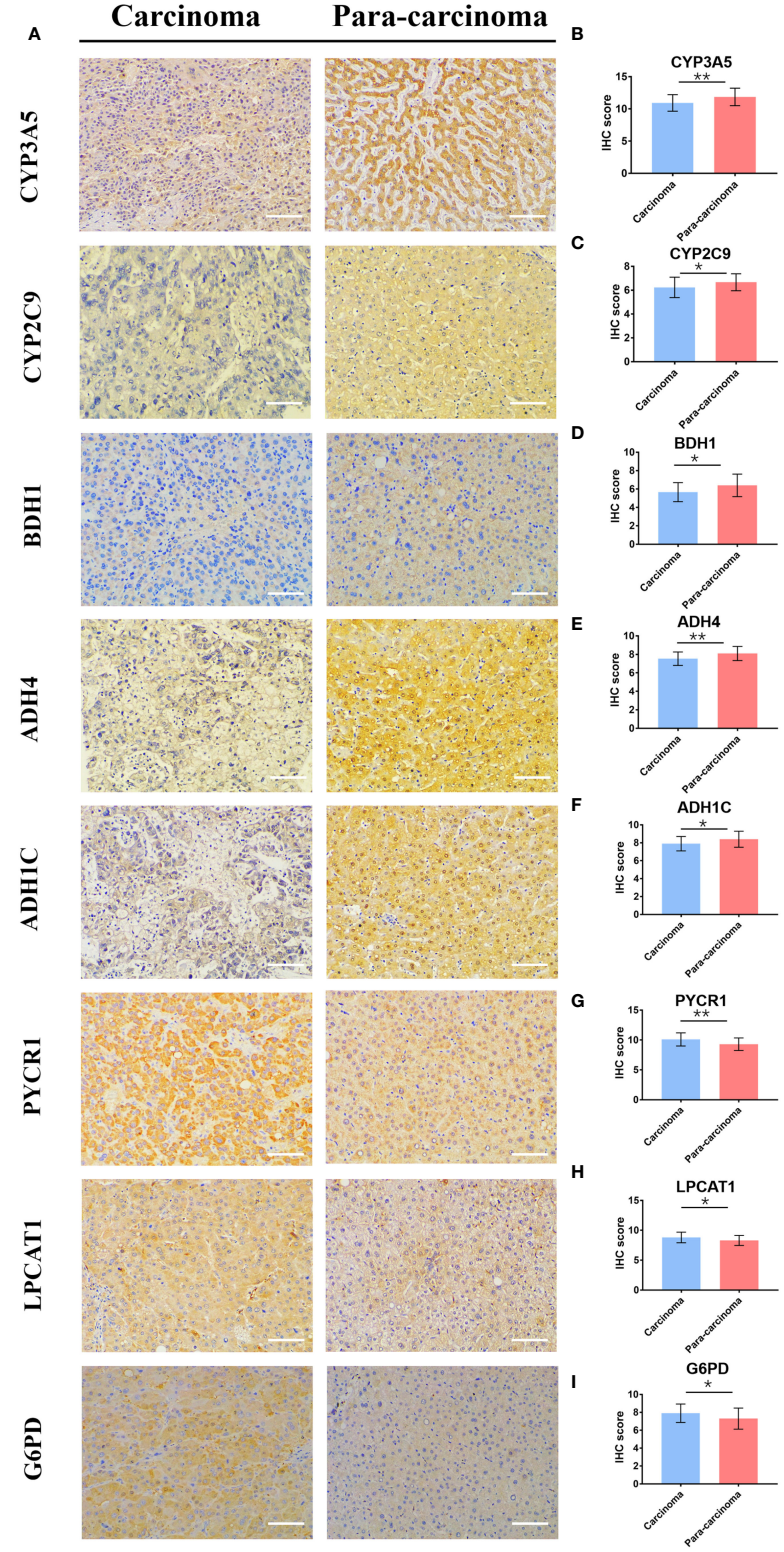
The differential expression of 8 genes in cancer and matched normal tissues. **(A)** Representative image of immunohistochemistry staining and **(B–I)** immunohistochemical staining scores. (× 200, scale bar = 100 µm). Data are presented as the means ± SD. *p < 0.05 and **p < 0.01.

To further verify the bioinformatics analysis results, we collected both HCC and matched para-carcinoma tissues for IHC. Compared with normal tissue, the PYCR1, LPCAT1, and G6PD significantly expressed more in HCC tissue, while CYP3A5, CYP2C9, BDH1, ADH4, and ADH1C expressed less (**Figure 8A**), and the expression of RRM2, PTGES, and HK2 has no significant difference between two tissues (**Figure S4A**). The statistical analysis results are shown in **Figures 8B–I** and **Figures S4B–D**. Most of these results were consistent with our prognostic model, further indicating that the gene-based classifier had great value in predicting the mortality for HCC patients.

### The DEMGs-Based Risk Score Outperforms Other HCC Prognostic Factors

**Figure 9:**
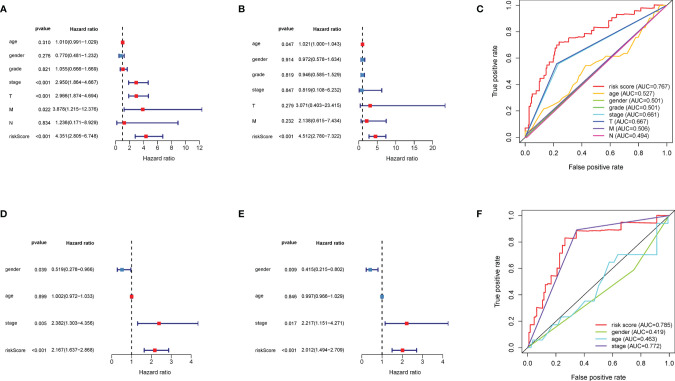
Comparison of prediction accuracy among the classifiers and other factors. The **(A)** univariate Cox analysis and **(B)** multivariate Cox analysis of DEMG-based risk score and other factors in TCGA. **(C)** The DEMG-based risk score was more accurate with an AUC of 0.767 in TCGA. The **(D)** univariate Cox analysis and **(E)** multivariate Cox analysis of risk score and other factors in LIRI_JP. **(F)** The DEMG-based risk score was more accurate with an AUC of 0.785 in LIRI_JP. DEMGs, differentially expressed metabolic genes; AUC, area under the curve.

For identifying the clinical significance of the DEMG-based survival-predictor model, we conducted the univariate Cox analysis in TCGA. The results indicated that tumor stage, T classification, M classification, and risk score were correlated with the survival rates (*p* < 0.05) (**Figure 9A**). Moreover, in multivariate Cox analysis, the risk score was significant (*p* < 0.01) while other factors were not associated with OS (*p* > 0.05) (**Figure 9B**). More importantly, the time-dependent ROC curves suggested that the DEMG-based risk score with an AUC of 0.767 could predict mortality more accurately than other HCC prognostic factors: age (AUC = 0.527), gender (AUC = 0.501), grade (AUC = 0.501), stage (AUC = 0.661), T (AUC = 0.667), N (AUC = 0.494), and M (AUC = 0.506) (**Figure 9C**). In LIRI-JP, we acquired the same results (**Figures 9D–F**).

## Discussion

As the center of human metabolism, the liver is engaged in the metabolic interchange of water-soluble and lipid metabolites all the time; no other organ can match the metabolic rate of the liver (30). Moreover, the biological processes of toxic substance decomposition and metabolism processes, P450 pathway, p53 pathway, and alcohol dehydrogenase activity have been reported to be related to HCC in previous studies (31–33). Thus, metabolism pathways in the liver may provide a way to predict the prognosis of HCC (34).

In this study, we divided HCC patients into two groups by analyzing the activation of metabolic pathways (**Figures 2E, F**). The two groups mainly showed significant differences in expressions in 10 metabolic pathways, such as purine metabolism, pyrimidine metabolism, and fructose and mannose metabolism. Tumor cells generate energy by glycolysis despite the presence of sufficient oxygen to support the proliferation and differentiation of cancer cells, which is called the Warburg effect (35). Glutamines, which provide the nitrogen required for the biosynthesis of purine and pyrimidine nucleotides, are also needed in the survival and growth of tumor cells (35). These studies are consistent with our conclusions, suggesting that the Metabolism_L subtype with a lower expression of purine metabolic, pyrimidine metabolic, fructose and mannose metabolic, and amino sugar and nucleotide sugar metabolic pathways will likely have a better survival prognosis (**Figures 2F, I**). The two subtypes also showed significant differences on taurine and hypotaurine metabolism, retinol metabolism, and fatty acid metabolism. Free fatty acid was confirmed as an independent risk factor for cancer (36), and statin can improve survival outcomes and increases overall survival (37–39). Furthermore, the retinol and retinal are also associated with the diagnosis and prognosis of HCC (16). In brief, the metabolic pathways selected in the current study were all related to survival and proliferation of tumors.

Then, we analyzed mutated genes and tumor immunity in two subtypes, respectively. **Figure 3** shows that the mutation rate of the *TP53* gene was significantly different in Metabolism_H and Metabolism_L, and Metabolism_H expressed more *TP53*. The wild-type *TP53* protein plays an important role in apoptosis after DNA damage and in cell cycle regulation (40). However, the mutant *TP53* protein loses its wild-type function and accumulates in the nucleus, which is considered to be a highly specific marker of malignant tumors (41). Similarly, *TP53* also plays an important role in HCC (42, 43). Mutant *TP53* proteins simultaneously lose their tumor-suppressive functions and obtain new capacities to advance tumorigenesis (44). Ling et al. (45) indicated that HCC patients with non-functional mutant genes of *TP53* tended to have a worse survival prognosis. CNV analysis suggested that the two HCC subtypes were different in chromosomal structural variation. As we have mentioned, several genes promoting the development of liver cancer, such as SCAMP3 and CCT3 (46), amplified more frequently in Metabolism_H. However, some gene expressions were negatively correlated with methylation. For instance, as shown in **Figure 4**, the levels of methylation of the MRG PDK4 were higher in Metabolism_H, and low expression of PDK4 promoted proliferation and metastasis of HCC (47). In conclusion, these findings explain why survival rates in Metabolism_H are lower. Interestingly, the two subtypes also showed significant differences in tumor immunity, and the immune-related pathways interacted with metabolic pathways. Some studies have proved that some immune cells associate with the prognosis of HCC, such as CD8+ T cells, regulatory T cells (Tregs), and B cells (48). HCC produced lactic and carbonic acids excessively by exacerbating glycolysis to change the tumor immunity microenvironment (49). **Figure S1** shows that Metabolism_L contained more tumor cells and less immune cells compared with Metabolism_H. Recent studies reported that immune cell infiltration could affect the prognosis of HCC and intratumoral infiltration by dendritic cells and neutrophils may result in poor prognosis in HCC patients (50, 51). Neutrophils not only were involved in the activation and regulation of immune cells, but also promoted the progression of HCC by releasing cytokines (52). Moreover, macrophages, especially M2 macrophages, contributed to the poor prognosis of HCC (53), whose infiltration within the tumor microenvironment could facilitate tumor growth, angiogenesis, invasion, as well as metastasis (54). According to our results, tumor tissue of Metabolism_H contained more M2 macrophages and neutrophils. Conversely, tumor tissue of Metabolism_H contained less CD8 T cells, which were the primary cytotoxic lymphocytes exerting antitumor effects (55). Although *p*-values of M2 macrophages, neutrophils, and CD8 T cells were less than 0.05 in only one of the two databases, which may be due to insufficient data, the difference in the two immune cells was consistent between the two databases. Moreover, a lower proportion of CD8 T cells indicated an immune-suppressive state in Metabolism_H, and the Metabolism_H subtype had higher mRNAsi and higher expressions of immune checkpoint-related genes such as PD-L1 (56, 57). Therefore, it was no surprise that the Metabolism_H had a lower survival rate.

According to the LASSO regression method, we determined 11 DEMGs: RRM2, PYCR1, PTGES, LPCAT1, HK2, G6PD, CYP3A5, CYP2C9, BDH1, ADH4, and ADH1C. Reports have indicated that most of the 11 DEMGs were closely related to the OS of tumor (58–61). Among the 11 DEMGs, PYCR1 plays a vital part in the promotion of HCC cell proliferation by increasing proline biosynthesis effectively (62). Additionally, LPCAT1 participates in cell proliferation, migration, and invasion by modulating phospholipid composition, in HCC (63). In our study, we divided the HCC patients into two groups based on the 11-DEMG-based classification in TCGA. Then, we verified the correctness of this grouping method in TCGA. The two groups all showed significantly different survival rates in TCGA and LIRI-JP. Moreover, the differential expression of the 11 genes in cancer and matched normal tissues was observed by immunohistochemistry staining. Compared with normal tissues, 8 genes showed remarkable differential expression between cancer tissues and normal tissues, while 3 genes revealed no significant differences. The limitations may come from differential expression patterns of genes, population differences, or statistical noise, and require more experiments to be verified. In addition, when compared with the clinicopathological risk factors, the 11-DEMG-based risk score was better at predicting survival in both TCGA and LIRI-JP, which was the highlight of this study. There is absolutely no doubt that our 11-DEMG-based classifiers possessed their own unique prediction. When the classifiers are combined with clinicopathological risk factors, it would provide a more accurate prediction for OS at different times for HCC patients. Therefore, the DEMG-based survival-predictor model has shown a favorable effect on survival prediction, which will contribute to therapeutic decision-making.

However, there are several limitations in this study. Firstly, we found that tumor metabolism was associated with tumor immunity, but, regrettably, this study mainly focused on the association between the MRGs and the OS of HCC. It will be interesting to combine metabolic genes with immune genes to predict HCC OS in the future. Secondly, this study was a retrospective study utilizing the TCGA and LIRI-JP databases. Therefore, more prospective studies were still needed. Third, if we could discover tumor biomarker detection in a more accessible blood sample, it would be more clinically valuable. Finally, a study at the single-cell level would be better in entangling the heterogeneity among the cells (64–66), which will be the subject of a future work.

## Conclusions

In summary, we identified two metabolism-based classifiers associated with OS in HCC and confirmed that the differences in survival rates in the two clusters may be related to mutated genes and tumor immunology. According to the LASSO regression method, we determined 11 DEMGs. Notably, the DEMG-based survival-predictor model could accurately predict the OS of HCC patients, and the results may contribute to the development of individual therapy.

## Data Availability Statement

The datasets used during the current study are available from the corresponding author on reasonable request.

## Ethics Statement

This study was reviewed and approved by the Medical Ethical Committee of the First Affiliated Hospital of Wenzhou Medical University (2021-R084).

## Author Contributions

ZZ and YS were responsible for study conception and design. TY, LL, WH, and SW were responsible for data collection and analysis. TY, LC, and WH were responsible for drafting the manuscript. TY and ZZ were responsible for revision of the manuscript. All authors contributed to the article and approved the submitted version.

## Conflict of Interest

The authors declare that the research was conducted in the absence of any commercial or financial relationships that could be construed as a potential conflict of interest.

## Publisher’s Note

All claims expressed in this article are solely those of the authors and do not necessarily represent those of their affiliated organizations, or those of the publisher, the editors and the reviewers. Any product that may be evaluated in this article, or claim that may be made by its manufacturer, is not guaranteed or endorsed by the publisher.

## Supplementary Material

Supplementary Figure 1 Analysis of Copy number variation. Copy number variation in **(A)** Metabolism_H and **(B)** Metabolism_L; **(C)** Focal copy number alterations in several genes.

Supplementary Figure 2 Different tumor immunoinfiltration between Metabolism_H and Metabolism_L. **(A)** the two clusters showed significantly different Stromal Score in LIRI−JP (Kruskal–Wallis test), while no significantly different Stromal Score in TCGA (Kruskal–Wallis test); **(B)** Immune Score was higher in Metabolism_H (Kruskal–Wallis test) both in TCGA and LIRI−JP; **(C)** Tumor Purity was higher in Metabolism_L (Kruskal–Wallis test) both in TCGA and LIRI−JP; **(D)** ESTIMATE Score was higher in Metabolism_H (Kruskal–Wallis test) both in TCGA and LIRI−JP. *P < 0.05, **P < 0.01, ***P < 0.001.

Supplementary Figure 3 The immune checkpoints related gene expression and the cancer stemness analyses. **(A)** PD-L1, CTLA-4 and TIM-3 expressed more in Metabolism_H; **(B)** mRNA expression-based stemness index (mRNAsi) was higher in the Metabolism_H. ***P < 0.001.

Supplementary Figure 4 The expression of 3 genes in cancer and matched normal tissues. **(A)** Representative image of immunohistochemistry staining and **(B–D)** immunohistochemical staining scores. (× 200, Scale bar = 100 µm). Data are presented as the means ± SD. ns, not significant.

## Abbreviations

HCC, Hepatocellular carcinoma; TCGA, The cancer genome atlas; ICGC, International cancer genome consortium; DEMGs, Differentially expressed metabolic genes; LASSO, Least absolute shrinkage and selection operator; tdROC, Time-dependent receiver operating characteristics; OS, Overall Survival; CNV, Copy number variation; TACE, Transarterial chemotherapy with embolization; ssGSEA, Single-sample gene-set enrichment analysis; AUC, Area under the curve; IHC, Immunohistochemistry.
