# Supplementary material for: Novel Prognostic Signatures of Hepatocellular Carcinoma Based on Metabolic Pathway Phenotypes
Source: Front Oncol. 2022 May 23;12:863266. doi: 10.3389/fonc.2022.863266 (PMC9168273; doi:10.3389/fonc.2022.863266)
Supplement: Supplementary Figure 1 — Analysis of Copy number variation. Copy number variation in (A) Metabolism_H and (B) Metabolism_L; (C) Focal copy number alterations in several genes. [file DataSheet_1.zip › Supplementary materials-revision/Table S1-revision.docx]

**Table S1. Clinical and pathological information of the thirty HCC patients.**

| **Parameter** | **Number of cases (%)** |
| --- | --- |
| **Gender**  Male  Female  **Age**  ≥60years  ＜60years  **Staging**  I+II  III+IV  **Hepatitis B status**  Positive  Negative  **AFP**  Positive  Negative  **Microvascular invasion**  Yes  No  **Differentiation**  High  Median  Low  **Largest tumor diameter**  ≥5cm  ＜5cm | 26 (86.7)  4 (13.3)  10 (33.3)  20 (66.7)  11 (36.7)  19 (63.3)  25 (83.3)  5 (16.7)  18 (60.0)  12 (40.0)  16 (53.3)  14 (46.7)  1 (3.3)  11 (36.7)  18 (60.0)  8 (26.7)  22 (73.3) |
