# Supplementary material for: Novel Prognostic Signatures of Hepatocellular Carcinoma Based on Metabolic Pathway Phenotypes
Source: Front Oncol. 2022 May 23;12:863266. doi: 10.3389/fonc.2022.863266 (PMC9168273; doi:10.3389/fonc.2022.863266)
Supplement: Supplementary Figure 1 — Analysis of Copy number variation. Copy number variation in (A) Metabolism_H and (B) Metabolism_L; (C) Focal copy number alterations in several genes. [file DataSheet_1.zip › Supplementary materials-revision/Table S4-revision.docx]

**Table S4. Gene sets enriched in the two phenotypes**

| **MSigDB collection** | **Phenotype** | **Gene set name** | **NES** | **NOM p-val** | **FDR q-val** |
| --- | --- | --- | --- | --- | --- |
| c2.cp.kegg.v7.0.symbols.gmt | Metabolism_H | KEGG_VIBRIO_CHOLERAE_INFECTION | 2.01 | 0 | 0.0077547 |
|  |  | KEGG_PATHOGENIC_ESCHERICHIA_COLI_INFECTION | 2.01 | 0 | 0.0103396 |
|  |  | KEGG_EPITHELIAL_CELL_SIGNALING_IN_HELICOBACTER_PYLORI_INFECTION | 1.95 | 0 | 0.0134062 |
|  |  | KEGG_PURINE_METABOLISM | 1.96 | 0 | 0.0139986 |
|  |  | KEGG_RNA_DEGRADATION | 2.02 | 0 | 0.0146175 |
|  |  | KEGG_PYRIMIDINE_METABOLISM | 1.96 | 0 | 0.0153646 |
|  |  | KEGG_OOCYTE_MEIOSIS | 1.92 | 0 | 0.0186927 |
|  |  | KEGG_FC_GAMMA_R_MEDIATED_PHAGOCYTOSIS | 1.91 | 0 | 0.019383 |
|  |  | KEGG_RENAL_CELL_CARCINOMA | 1.86 | 0 | 0.0203526 |
|  |  | KEGG_SPLICEOSOME | 2.03 | 0 | 0.0205406 |
|  |  | KEGG_DNA_REPLICATION | 1.87 | 0 | 0.0218329 |
|  |  | KEGG_HUNTINGTONS_DISEASE | 1.86 | 0.0063425 | 0.0220579 |
|  |  | KEGG_ENDOCYTOSIS | 1.85 | 0 | 0.0228211 |
|  |  | KEGG_VASOPRESSIN_REGULATED_WATER_REABSORPTION | 1.88 | 0 | 0.0231722 |
|  |  | KEGG_PATHWAYS_IN_CANCER | 1.87 | 0 | 0.0232142 |
|  |  | KEGG_P53_SIGNALING_PATHWAY | 1.8 | 0.0044444 | 0.0247649 |
|  |  | KEGG_CELL_CYCLE | 1.88 | 0 | 0.0252 |
|  |  | KEGG_LYSOSOME | 1.79 | 0.0065359 | 0.0256461 |
|  |  | KEGG_BASE_EXCISION_REPAIR | 1.8 | 0.0020661 | 0.0257174 |
|  |  | KEGG_ADHERENS_JUNCTION | 1.79 | 0.004415 | 0.0262475 |
|  |  | KEGG_CYTOSOLIC_DNA_SENSING_PATHWAY | 1.8 | 0.0058824 | 0.0267061 |
|  |  | KEGG_REGULATION_OF_ACTIN_CYTOSKELETON | 1.81 | 0 | 0.0272549 |
|  |  | KEGG_NOD_LIKE_RECEPTOR_SIGNALING_PATHWAY | 1.8 | 0.0044843 | 0.0273853 |
|  |  | KEGG_NUCLEOTIDE_EXCISION_REPAIR | 1.81 | 0.0020619 | 0.02776 |
|  |  | KEGG_VEGF_SIGNALING_PATHWAY | 1.77 | 0 | 0.0285976 |
|  |  | KEGG_NOTCH_SIGNALING_PATHWAY | 1.81 | 0.0021231 | 0.028895 |
|  |  | KEGG_HOMOLOGOUS_RECOMBINATION | 1.76 | 0.0020408 | 0.0291214 |
|  |  | KEGG_LEISHMANIA_INFECTION | 1.82 | 0.0067114 | 0.0291229 |
|  |  | KEGG_SNARE_INTERACTIONS_IN_VESICULAR_TRANSPORT | 1.76 | 0.0021008 | 0.0292703 |
|  |  | KEGG_PROTEIN_EXPORT | 1.77 | 0.0105485 | 0.0293398 |
|  |  | KEGG_AXON_GUIDANCE | 1.76 | 0.0022472 | 0.0296042 |
|  |  | KEGG_AMINOACYL_TRNA_BIOSYNTHESIS | 1.77 | 0.0080808 | 0.029733 |
|  |  | KEGG_BLADDER_CANCER | 1.81 | 0.0022472 | 0.0298054 |
|  |  | KEGG_UBIQUITIN_MEDIATED_PROTEOLYSIS | 1.82 | 0.0042105 | 0.0299697 |
|  |  | KEGG_CHRONIC_MYELOID_LEUKEMIA | 1.75 | 0.0085106 | 0.0301915 |
|  |  | KEGG_PROTEASOME | 1.82 | 0.0060484 | 0.0303952 |
|  |  | KEGG_PROGESTERONE_MEDIATED_OOCYTE_MATURATION | 1.75 | 0 | 0.0309562 |
|  |  | KEGG_T_CELL_RECEPTOR_SIGNALING_PATHWAY | 1.74 | 0.0114155 | 0.0311308 |
|  |  | KEGG_GLYCEROPHOSPHOLIPID_METABOLISM | 1.74 | 0 | 0.0313787 |
|  |  | KEGG_SMALL_CELL_LUNG_CANCER | 1.74 | 0.0043197 | 0.0315851 |
|  |  | KEGG_NEUROTROPHIN_SIGNALING_PATHWAY | 1.73 | 0.0133038 | 0.0322764 |
|  |  | KEGG_GAP_JUNCTION | 1.73 | 0 | 0.032686 |
|  |  | KEGG_PANCREATIC_CANCER | 1.71 | 0.021692 | 0.0351955 |
|  |  | KEGG_COLORECTAL_CANCER | 1.72 | 0.0152838 | 0.0353598 |
|  |  | KEGG_WNT_SIGNALING_PATHWAY | 1.71 | 0.0023364 | 0.035596 |
|  |  | KEGG_DORSO_VENTRAL_AXIS_FORMATION | 1.71 | 0.0044743 | 0.0366983 |
|  |  | KEGG_APOPTOSIS | 1.7 | 0.010917 | 0.0367452 |
|  |  | KEGG_MTOR_SIGNALING_PATHWAY | 1.69 | 0.0086957 | 0.038799 |
|  |  | KEGG_GNRH_SIGNALING_PATHWAY | 1.69 | 0.0022624 | 0.0388003 |
|  |  | KEGG_ALZHEIMERS_DISEASE | 1.68 | 0.0246637 | 0.0410621 |
|  |  | KEGG_BASAL_TRANSCRIPTION_FACTORS | 1.68 | 0.012685 | 0.0414704 |
|  |  | KEGG_B_CELL_RECEPTOR_SIGNALING_PATHWAY | 1.66 | 0.0184758 | 0.0425396 |
|  |  | KEGG_TIGHT_JUNCTION | 1.65 | 0.0046083 | 0.043198 |
|  |  | KEGG_GLYCOSPHINGOLIPID_BIOSYNTHESIS_LACTO_AND_NEOLACTO_SERIES | 1.66 | 0.0066079 | 0.0432859 |
|  |  | KEGG_RIBOSOME | 1.65 | 0.0080808 | 0.0434925 |
|  |  | KEGG_RNA_POLYMERASE | 1.67 | 0.016 | 0.0436869 |
|  |  | KEGG_GLYCOSAMINOGLYCAN_BIOSYNTHESIS_HEPARAN_SULFATE | 1.67 | 0.0111857 | 0.0438465 |
|  |  | KEGG_RIG_I_LIKE_RECEPTOR_SIGNALING_PATHWAY | 1.64 | 0.0242915 | 0.043876 |
|  |  | KEGG_FOCAL_ADHESION | 1.66 | 0.0296804 | 0.0438784 |
|  |  | KEGG_CHEMOKINE_SIGNALING_PATHWAY | 1.65 | 0.0374707 | 0.0439172 |
|  |  | KEGG_MISMATCH_REPAIR | 1.66 | 0.0101626 | 0.0439285 |
|  |  | KEGG_LONG_TERM_DEPRESSION | 1.65 | 0.0071429 | 0.0439578 |
|  |  | KEGG_NATURAL_KILLER_CELL_MEDIATED_CYTOTOXICITY | 1.64 | 0.034632 | 0.044166 |
|  |  | KEGG_MAPK_SIGNALING_PATHWAY | 1.64 | 0.0047847 | 0.0442922 |
|  |  | KEGG_FC_EPSILON_RI_SIGNALING_PATHWAY | 1.67 | 0.0069124 | 0.0444845 |
|  |  | KEGG_N_GLYCAN_BIOSYNTHESIS | 1.63 | 0.0294118 | 0.0459524 |
|  |  | KEGG_ERBB_SIGNALING_PATHWAY | 1.61 | 0.032967 | 0.0510728 |
|  |  | KEGG_GLYCOSAMINOGLYCAN_BIOSYNTHESIS_CHONDROITIN_SULFATE | 1.61 | 0.0243902 | 0.0512614 |
|  |  | KEGG_NON_SMALL_CELL_LUNG_CANCER | 1.58 | 0.0323974 | 0.0578315 |
|  |  | KEGG_TOLL_LIKE_RECEPTOR_SIGNALING_PATHWAY | 1.59 | 0.0323974 | 0.0580724 |
|  |  | KEGG_LEUKOCYTE_TRANSENDOTHELIAL_MIGRATION | 1.58 | 0.0261283 | 0.058339 |
|  |  | KEGG_CARDIAC_MUSCLE_CONTRACTION | 1.59 | 0.0180879 | 0.0584259 |
|  |  | KEGG_MELANOMA | 1.58 | 0.0117925 | 0.0586013 |
|  |  | KEGG_MELANOGENESIS | 1.58 | 0.0048426 | 0.0591237 |
|  |  | KEGG_GLIOMA | 1.57 | 0.0331126 | 0.0609162 |
|  |  | KEGG_REGULATION_OF_AUTOPHAGY | 1.55 | 0.0359712 | 0.0679084 |
|  |  | KEGG_SPHINGOLIPID_METABOLISM | 1.55 | 0.0416667 | 0.0688115 |
|  |  | KEGG_LONG_TERM_POTENTIATION | 1.55 | 0.0255814 | 0.0689439 |
|  |  | KEGG_FRUCTOSE_AND_MANNOSE_METABOLISM | 1.53 | 0.0263158 | 0.0772869 |
|  |  | KEGG_RIBOFLAVIN_METABOLISM | 1.52 | 0.0382023 | 0.0781399 |
|  |  | KEGG_ETHER_LIPID_METABOLISM | 1.51 | 0.028777 | 0.0799552 |
|  |  | KEGG_HEDGEHOG_SIGNALING_PATHWAY | 1.43 | 0.0454545 | 0.1141422 |
|  | Metabolism_L | KEGG_RETINOL_METABOLISM | -2.4 | 0 | 0 |
|  |  | KEGG_DRUG_METABOLISM_CYTOCHROME_P450 | -2.3 | 0 | 0 |
|  |  | KEGG_FATTY_ACID_METABOLISM | -2.2 | 0 | 0 |
|  |  | KEGG_PPAR_SIGNALING_PATHWAY | -2.2 | 0 | 4.41E-05 |
|  |  | KEGG_METABOLISM_OF_XENOBIOTICS_BY_CYTOCHROME_P450 | -2.2 | 0 | 5.15E-05 |
|  |  | KEGG_PEROXISOME | -2.2 | 0 | 6.17E-05 |
|  |  | KEGG_GLYCINE_SERINE_AND_THREONINE_METABOLISM | -2.2 | 0 | 7.72E-05 |
|  |  | KEGG_VALINE_LEUCINE_AND_ISOLEUCINE_DEGRADATION | -2.2 | 0 | 1.01E-04 |
|  |  | KEGG_TRYPTOPHAN_METABOLISM | -2.1 | 0 | 1.37E-04 |
|  |  | KEGG_PRIMARY_BILE_ACID_BIOSYNTHESIS | -2.1 | 0 | 1.86E-04 |
|  |  | KEGG_BUTANOATE_METABOLISM | -2.1 | 0 | 2.31E-04 |
|  |  | KEGG_PROPANOATE_METABOLISM | -2.1 | 0 | 2.41E-04 |
|  |  | KEGG_STEROID_HORMONE_BIOSYNTHESIS | -2.1 | 0 | 3.09E-04 |
|  |  | KEGG_BETA_ALANINE_METABOLISM | -2 | 0 | 8.93E-04 |
|  |  | KEGG_COMPLEMENT_AND_COAGULATION_CASCADES | -1.9 | 0.0019569 | 0.0014594 |
|  |  | KEGG_ASCORBATE_AND_ALDARATE_METABOLISM | -1.9 | 0.0054446 | 0.0019773 |
|  |  | KEGG_DRUG_METABOLISM_OTHER_ENZYMES | -1.9 | 0 | 0.0024769 |
|  |  | KEGG_TYROSINE_METABOLISM | -1.9 | 0 | 0.0043604 |
|  |  | KEGG_LINOLEIC_ACID_METABOLISM | -1.8 | 0.0053381 | 0.0044243 |
|  |  | KEGG_HISTIDINE_METABOLISM | -1.8 | 0 | 0.005057 |
|  |  | KEGG_ARGININE_AND_PROLINE_METABOLISM | -1.8 | 0.0017637 | 0.0074878 |
|  |  | KEGG_PYRUVATE_METABOLISM | -1.7 | 0.0160428 | 0.0140957 |
|  |  | KEGG_PENTOSE_AND_GLUCURONATE_INTERCONVERSIONS | -1.7 | 0.0198915 | 0.0172672 |
|  |  | KEGG_ALANINE_ASPARTATE_AND_GLUTAMATE_METABOLISM | -1.7 | 0.010929 | 0.019917 |
|  |  | KEGG_PHENYLALANINE_METABOLISM | -1.7 | 0.0176056 | 0.0267858 |
|  |  | KEGG_BIOSYNTHESIS_OF_UNSATURATED_FATTY_ACIDS | -1.6 | 0.0353818 | 0.0302983 |
|  |  | KEGG_STARCH_AND_SUCROSE_METABOLISM | -1.6 | 0.0158451 | 0.0313951 |
|  |  | KEGG_ARACHIDONIC_ACID_METABOLISM | -1.6 | 0.0169779 | 0.0474438 |
