# Supplementary material for: Novel Prognostic Signatures of Hepatocellular Carcinoma Based on Metabolic Pathway Phenotypes
Source: Front Oncol. 2022 May 23;12:863266. doi: 10.3389/fonc.2022.863266 (PMC9168273; doi:10.3389/fonc.2022.863266)
Supplement: Supplementary Figure 1 — Analysis of Copy number variation. Copy number variation in (A) Metabolism_H and (B) Metabolism_L; (C) Focal copy number alterations in several genes. [file DataSheet_1.zip › Supplementary materials-revision/Table S5-revision.docx]

**Table S5. The detailed information of differentially expressed metabolic genes for constructing the prognostic signature**

| **Gene name** | **ENSG_ ID** | **Gene_type** | **bp** | **Chromosome** | **β** |
| --- | --- | --- | --- | --- | --- |
| ADH1C | ENSG00000248144.1 | Polymorphic pseudogene | 1740 | Chromosome 4: 100,257,649-100,274,184 | -3E-04 |
| ADH4 | ENSG00000198099.4 | Protein coding | 1993 | Chromosome 4: 100,044,808-100,078,949 | -2E-04 |
| BDH1 | ENSG00000161267.7 | Protein coding | 3455 | Chromosome 3: 197,236,654-197,300,194 | -0.002 |
| CYP2C9 | ENSG00000138109.9 | Protein coding | 1847 | Chromosome 10: 96,698,415-96,749,147 | -6E-04 |
| CYP3A5 | ENSG00000106258.9 | Protein coding | 1720 | Chromosome 7: 99,245,817-99,277,621 | -0.002 |
| G6PD | ENSG00000160211.11 | Protein coding | 2631 | Chromosome X: 153,759,606-153,775,787 | 0.0082 |
| HK2 | ENSG00000159399.5 | Protein coding | 5772 | [Chromosome 2: 75,061,108-75,120,486](http://grch37.ensembl.org/Homo_sapiens/Location/View?db=core;g=ENSG00000159399;r=2:75061108-75120486) | 0.0154 |
| LPCAT1 | ENSG00000153395.5 | Protein coding | 3966 | Chromosome 5: 1,456,595-1,524,092 | 8E-05 |
| PTGES | ENSG00000148344.10 | Protein coding | 1774 | [Chromosome 9: 132,500,610-132,515,326](http://grch37.ensembl.org/Homo_sapiens/Location/View?db=core;g=ENSG00000148344;r=9:132500610-132515326;t=ENST00000340607) | 0.0005 |
| PYCR1 | ENSG00000183010.12 | Protein coding | 1890 | [Chromosome 17: 79,890,260-79,900,288](http://grch37.ensembl.org/Homo_sapiens/Location/View?db=core;g=ENSG00000183010;r=17:79890260-79900288) | 0.0006 |
| RRM2 | ENSG00000171848.9 | Protein coding | 3673 | Chromosome 2: 10,262,455-10,271,545 | 0.0177 |
