# Supplementary figures and images for: Novel Prognostic Signatures of Hepatocellular Carcinoma Based on Metabolic Pathway Phenotypes
Source: Front Oncol. 2022 May 23;12:863266. doi: 10.3389/fonc.2022.863266 (PMC9168273; doi:10.3389/fonc.2022.863266)

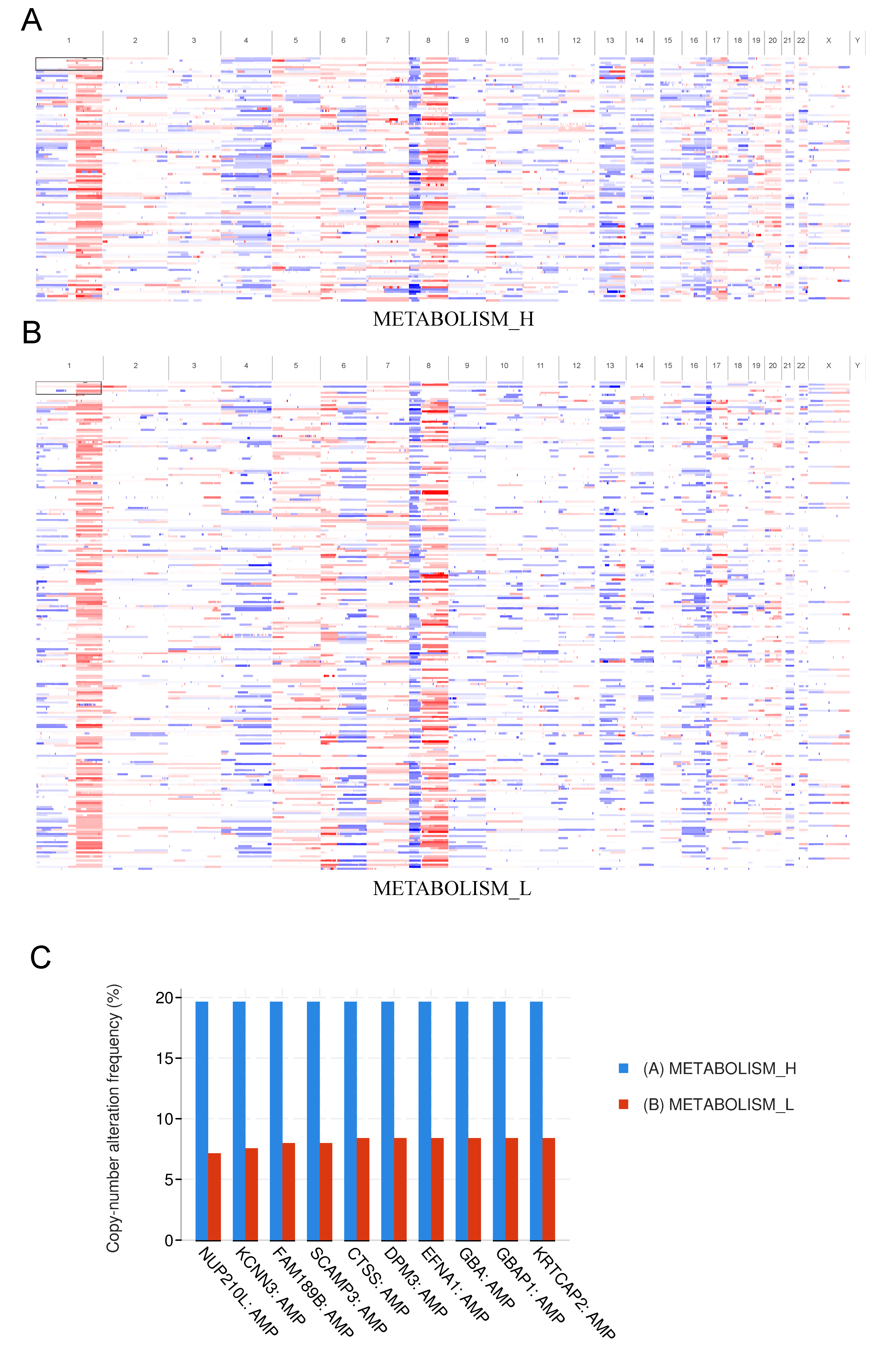

Supplement: Supplementary Figure 1 — Analysis of Copy number variation. Copy number variation in (A) Metabolism_H and (B) Metabolism_L; (C) Focal copy number alterations in several genes. [file DataSheet_1.zip › Supplementary materials-revision/Figure S1-revision.tif]

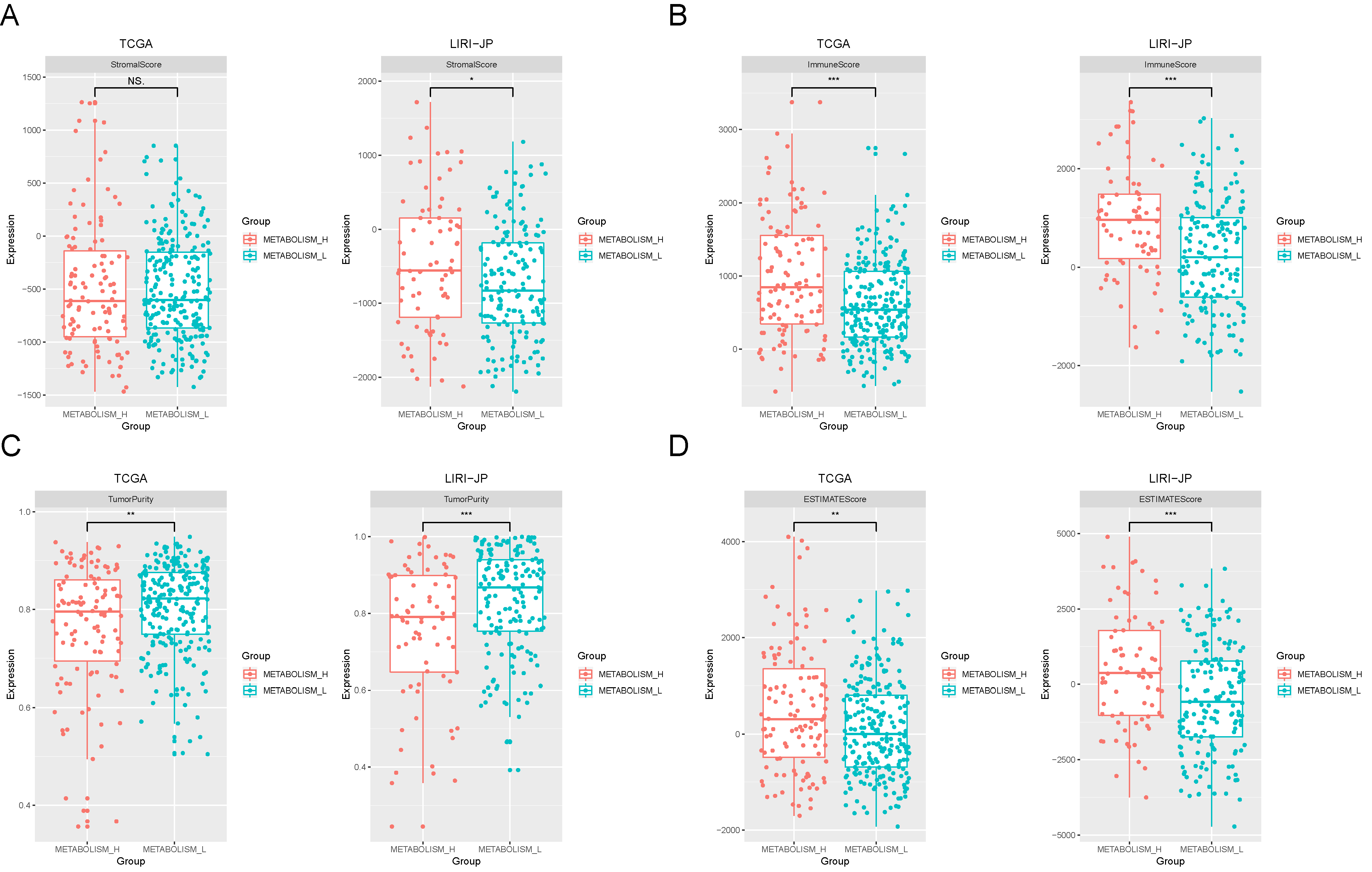

Supplement: Supplementary Figure 1 — Analysis of Copy number variation. Copy number variation in (A) Metabolism_H and (B) Metabolism_L; (C) Focal copy number alterations in several genes. [file DataSheet_1.zip › Supplementary materials-revision/Figure S2-revision.tiff]

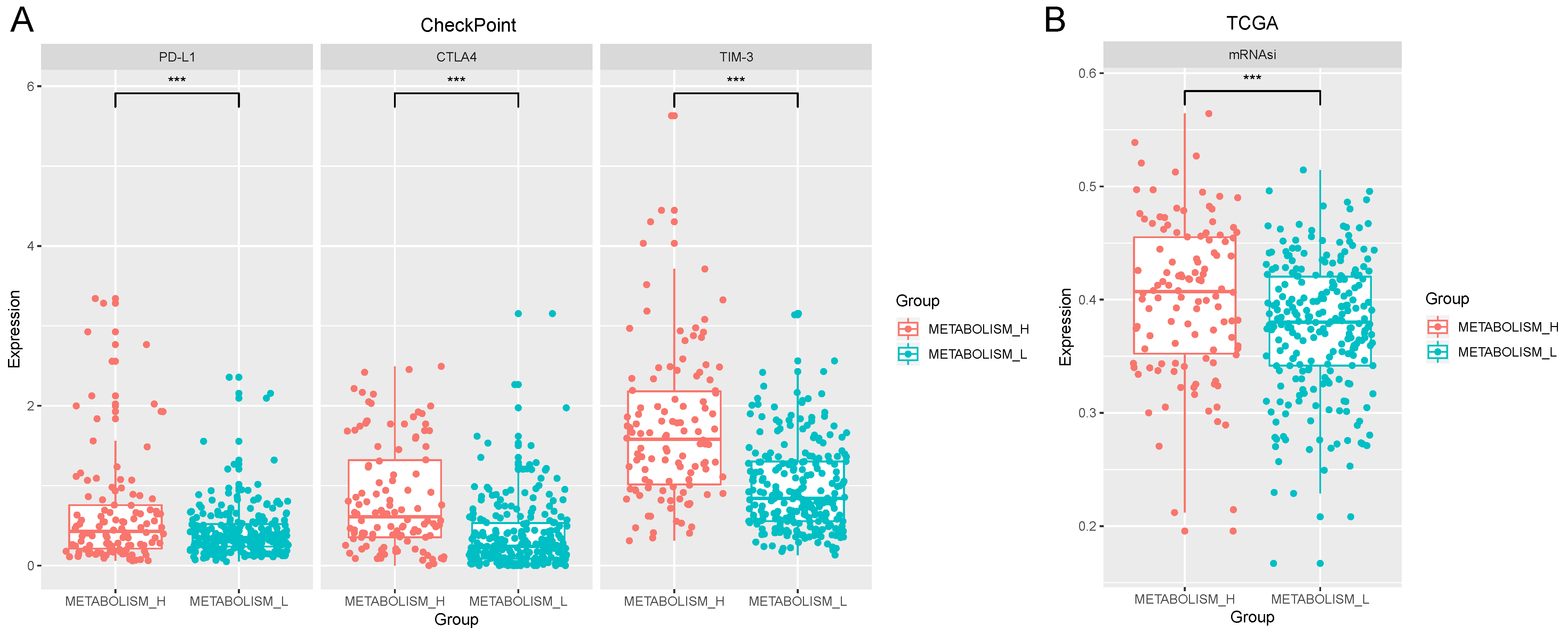

Supplement: Supplementary Figure 1 — Analysis of Copy number variation. Copy number variation in (A) Metabolism_H and (B) Metabolism_L; (C) Focal copy number alterations in several genes. [file DataSheet_1.zip › Supplementary materials-revision/Figure S3-revision.tiff]

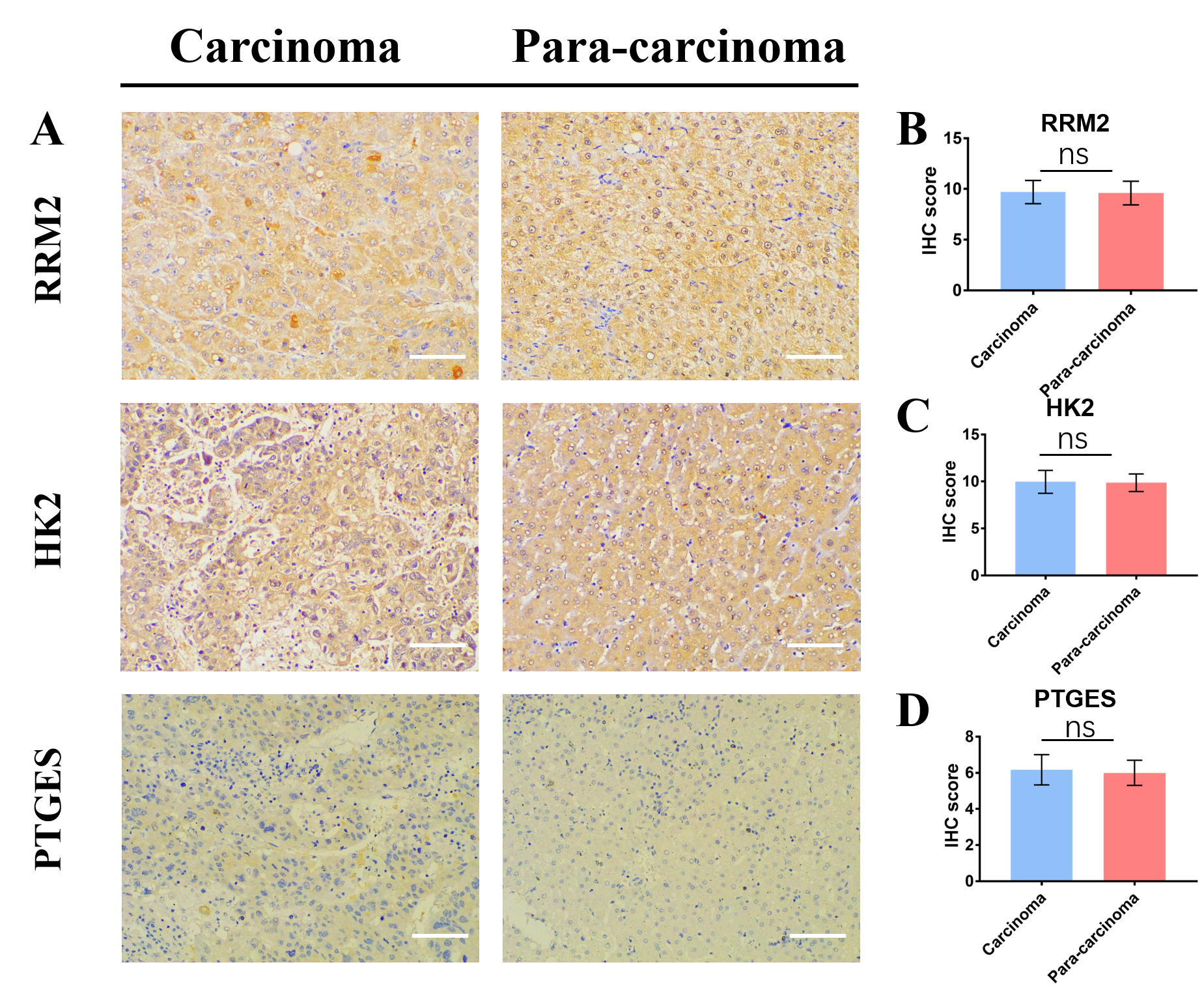

Supplement: Supplementary Figure 1 — Analysis of Copy number variation. Copy number variation in (A) Metabolism_H and (B) Metabolism_L; (C) Focal copy number alterations in several genes. [file DataSheet_1.zip › Supplementary materials-revision/Figure S4-revision.tif]
